# Evidence of target enhancement and distractor suppression in early visual areas

**DOI:** 10.3758/s13414-023-02673-w

**Published:** 2023-03-14

**Authors:** Julia Föcker, Anton L. Beer, Daphne Bavelier

**Affiliations:** 1 School of Psychology, College of Social Science, University of Lincoln, Lincoln, UK; 2 University of Regensburg, Institute of Psychology, Universitätsstr. 31, 93053 Regensburg, Germany; 3 University of Geneva, Brain and Learning Lab, Section of Psychology, Boulevard pont d’Arve 40, CH-1205 Geneva, Switzerland

**Keywords:** Attentional control, Retinotopic mapping, Target enhancement, Distractor suppression, Perceptual load

## Abstract

Although the mechanisms of target enhancement and distractor suppression have been investigated along the visual processing hierarchy, there remains some unknown as to the role of perceptual load on the competition between different task-related information as attention deployment is manipulated. We present an fMRI spatial cueing paradigm, in which 32 participants had to attend to either a left or a right hemifield location and to indicate the orientation of the target Gabor that was presented simultaneously to a noise patch distractor. Critically, the target could appear at either the cued, valid location or at the uncued, invalid location; in the latter, the noise patch distractor appeared at the cued location. Perceptual load was manipulated by the presence or absence of high-contrast Gabor patches close to the fixation cross, which acted as lateral masks. Behavioural results indicated that participants performed more efficiently in validly cued trials compared to invalidly cued trials and under low compared to high load. Enhancement effects for targets and suppression effects for noise patches were greater in early visual areas at high load, that is in the presence of lateral masks. These results are in line with the hypothesis that attention results in both target enhancement and distractor suppression, and that these effects are most marked under high perceptual load. Theoretical implications of these results for different models of attention are discussed.

## Introduction

The ability to focus on task-relevant information while ignoring distractors has been investigated in a broad range of behavioural, electrophysiological and brain-imaging paradigms (see Wöstmann et al., 2022, for a review). Whereas the neural mechanisms of target enhancement have been excessively investigated, there remains some debate about distractor suppression and, in particular, the neural mechanisms by which attention modulates the processing of distractors under different perceptual load conditions along the hierarchy of early visual cortical areas, such as V1, V2, V3 (Bahrami et al., 2007; O'Connor et al., 2002; Pinsk et al., 2004; Rees et al., 1997; Schwartz et al., 2005; Torralbo et al., 2016; Yi et al., 2004; see also Brockhoff et al., 2022, for a review).

### Neural mechanisms of target enhancement

Evidence of enhanced neural responses to attended compared to unattended stimuli (target enhancement) have been observed in animal and human studies at early processing stages, such as the lateral geniculate nucleus (LGN) and primary visual area (V1) as well as higher stages of the visual processing hierarchy, for example, the fusiform face area, the parahippocampal place area, the lateral occipital cortex, the superior parietal lobule, the frontal eye field and the supplementary eye field extending into the anterior cingulate cortex (Brefczynski & DeYoe, 1999; Chun & Turk-Browne, 2007; Corbetta & Shulman, 2002; Halassa & Kastner, 2017; Heinze et al., 1994; Kanwisher & Wojciulik, 2000; Kastner et al.*,* 1998; Murray & Wojciulik, 2004; O'Connor et al., 2002; Serences et al., 2004; Somers et al., 1999; Tootell et al., 1998; Wojciulik et al., 1998; for a review, see Beck & Kastner, 2014; Kastner & Ungerleider, 2000; Serences & Kastner, 2014). O’Connor and co-authors (2002) presented checkerboards in the left and right hemifield while participants were asked, in the attended condition, to direct their attention to the checkerboard and detect randomly occurring luminance changes of those checkerboards. In the unattended condition, participants were asked to perform a task at fixation. The blood-oxygenation-level-dependent (BOLD) response to the high contrast checkerboards was significantly increased when attended compared to when unattended in all early visual areas (O'Connor et al., 2002). Consistent with the hypothesis that attention “operates through top-down signals that are transmitted via corticocortical feedback connections in a hierarchical fashion” (p. 486, Kastner & Pinsk, 2004), attention effects observed in V1, V2, V3, V4, TEO and MT were reported to increase almost “gradually” from early to “more advanced processing levels along both the ventral and dorsal pathways of the visual cortex” (p. 486, Kastner & Pinsk, 2004). More generally, increased neural activity in response to attended stimuli has been interpreted as “neural gain control” (Hillyard et al., 1998). A large range of animal and human studies has shown that the amplitudes of sensory electrical responses are enlarged when attention is directed towards a stimulus and possibly suppressed when attention is oriented elsewhere (Hillyard & Anllo-Vento, 1998; see Hillyard et al., 1998).

Various experimental designs have been applied to investigate the principles of target enhancement, such as multiple object tracking (Howe et al., 2009), visual search (Adam & Serences, 2021), or Posner cueing designs (Doricchi et al., 2010). In the context of the multiple object-tracking task, two different attentional mechanisms have been suggested to explain target enhancement. According to the *“push-only”* mechanism, attention is “intentionally directed only toward targets and not toward distractors” (p. 2, Bettencourt & Somers, 2009; see Alvarez & Franconeri, 2007; Pylyshyn & Storm, 1988). This view suggests that attention acts as an amplification of target processing, leaving distractors unprocessed unless they intrude target selection. On the other hand, “*push-pull”* mechanisms hold that attention results in both enhancing target processing and suppressing the processing of distractor information (Pinsk et al., 2004; Posner et al., 1980; Somers et al., 1999; Yi et al., 2004). In this view, distractors automatically attract attention and thus may reduce target processing even if the distractors do not enter a target selection window. Active control mechanisms, such as the top-down deployment of attention, and/or passive pulls on attention, such as attentional capture, may “pull” attention away from distractors.

### The effect of load on target enhancement

Whereas the literature outlined above mainly focuses on target enhancement (higher neural signal to attended compared to unattended information), there are comparatively fewer functional MRI (fMRI) studies that address the question of how attention-based target enhancement might be modulated by load in early visual areas. The load theory of Lavie (1995) predicts that increasing perceptual load should lead to greater resource allocation at task-related locations, and concurrently reduced resources at distractor locations.

Steady-state visual evoked potentials (SSVEPs) together with event-related potentials (ERPs) have been analyzed to investigate target enhancement and distractor suppression under different load conditions (Handy & Mangun, 2000; Parks et al., 2013; Rauss et al., 2009, 2011, 2012; Rorden et al., 2008). In one experiment, participants performed a visual go/no-go task in the centre of the screen while irrelevant distractors were presented (Parks et al., 2013). In the low-load condition, targets could be distinguished from distractors by color, whereas in the high-load condition, both the features color and orientation were task relevant. Event-related potentials were recorded to the task-relevant stimuli presented at fixation while load was varied. The visual N1 (a negativity occurring around 100 ms after stimulus onset) was higher in the high-load compared to the low-load condition. This has been interpreted as an “enhancement in perceptual processing occurring as a result of the increased attentional demands required under high perceptual load, and potentially mediated by a top-down biasing signal” (p. 5. Parks et al., 2013). In line with the hypothesis that target enhancement is increased under high perceptual load, Handy and Mangun (2000) observed increased spatial attention effects in the time range of the P1 and N1 when perceptual load increased. Another study documented that cueing effects, or the behavioural performance to validly cued stimuli compared to invalidly cued stimuli, is enhanced when competing distractors are present on the screen compared to when they are absent (see Awh & Pashler, 2000). The authors suggested that “the primary effect of attention in these experiments is the exclusion of noise from distractor stimuli” (p. 842, Awh & Pashler, 2000).

To investigate the impact of multiple competing stimuli on target processing in visual brain areas, four different single stimuli were presented sequentially, one at a time, or simultaneously (Beck & Kastner, 2005; Beck & Kastner, 2007; Kastner et al., 1998; McMains & Kastner, 2010; McMains & Kastner, 2011). In the attended condition participants were asked to attend to the stimulus that was closest to the fixation point and count the occurrences of a target stimulus at that location, whereas in the unattended condition participants were asked to count letters at fixation while the same stimuli were presented in the periphery (Kastner et al., 1998). The neural target enhancement effect (difference between attended and unattended BOLD signal) was larger for simultaneously presented stimuli (when perceptual load was high) compared to sequential presentations, especially in area V4 and TEO. It has been suggested that the “magnitude of the attentional effect scaled with the magnitude of the suppressive interactions between stimuli, with the strongest reduction of suppression occurring in V4 and TEO” (p. 109, Kastner et al., 1998). To conclude, most studies report a neural and behavioural signal enhancement for targets under the high-load condition.

### Neural mechanisms of distractor suppression

Distractor suppression refers to the “ability to filter out distracting and task-irrelevant information” (p. 1, Wöstmann et al., 2022, for a review). Recently, it has been argued that the exact neural mechanisms of distractor suppression are unknown because of a lack of mutual consensus on how to study and explore distractor suppression as rather different experimental paradigms have been used to identify distractor suppression, and the exact definition of a distracting event also varies (see Wöstmann et al., 2022, for a review).

In a brain-imaging experiment increased perceptual load resulted in a gradual increase in activity within target-related areas along with a concomitant reduced activity in distractor-related areas, at least in V2 and V3 (Torralbo et al., 2016). In that same study, functional connectivity analyses further indicated that increased load led to increased functional connectivity between the BOLD V3 signal extracted from task-irrelevant distractor (checkerboard) and BOLD in the left inferior frontal gyrus, suggesting a down-regulation of distractor processing via frontal control (Torralbo et al., 2016). The majority of brain-imaging studies manipulating load have rather focused on the fate of distractors, and as reviewed below have documented that indeed distractor processing is reduced as load increases.

### The effect of load on distractor suppression

One of the first experimental designs to investigate suppression of *unattended irrelevant distractors* under *different load conditions* in humans used motion patterns (an optic flow pattern including dots moving radially toward the screen edge) as distractors and applied a central linguistic task, in which individuals were asked to press a key when a word was printed in upper case letter under the low-load condition, whereas they had to press a key whenever they saw a bisyllabic word under the high-load condition (Rees et al., 1997). The authors observed lower activation in the motion area MT in response to motion distractors, when a high-load central task was applied compared to a low-load central task. Other authors observed distractor effects in extrastriate or higher visual areas that were absent in early visual areas (V1, V2; see Pinsk et al., 2004). The authors interpreted these results as a load-dependent “push-pull” mechanism of selective attention operating at intermediate, but not early, processing stages of the visual-processing hierarchy (Pinsk et al., 2004).

Whereas Rees et al. (1997) and Pinsk et al. (2004) mainly observed distractor effects in extrastriate visual areas, Schwartz and co-authors (2005) identified distractor effects in early visual area V1. The authors presented a central visual search task under high- and low-load conditions, while participants were asked to ignore irrelevant checkerboards, which were presented in the lower visual field, either on the left side, right side, or bilaterally. In the control condition no checkerboard was presented. The BOLD signal in response to the checkerboard was reduced under the high-load condition compared to the low-load condition in V1, V2, V3, and V4. In order to investigate whether load effects lead to a “perceptual narrowing” of the visual information processing, such that visual information is mainly effectively processed in the centre by suppressing information at outer peripheral locations (tunnel vision), different retinotopic eccentricities, such as inner (2.8°) and outer (8.4°) peripheral eccentricities were defined. Contrary to the hypothesis that high attentional load at fixation produces “tunnel vision”, Schwartz and co-authors demonstrated that reduced activation in V1 in the high-load condition was mainly observed for inner peripheral eccentricities compared to outer peripheral eccentricities. These results have been interpreted as a “suppressive surround” mechanism, by which perceptual load at central fixation would impact areas *close* to the target compared to areas further in the periphery. This is in line with the push-pull mechanism demonstrated in neuro-physiological studies of attention, by which attentional effects are greatest when target and distractors fall within the same receptive field.

Different theoretical accounts have been considered to explain distractor suppression in the early visual cortex. The biased competition account (Desimone & Duncan, 1995) explains the increased processing of attended information and the suppression activity of unattended information by orienting attention to a specific location or a specific feature. The perceptual load theory (Lavie, 1995) further modulates this view by proposing that selection occurs early when perceptual difficulty (or load) is high, whereas under low perceptual difficulty (or load), distractors reach higher processing stages. Different methods have been applied to evaluate this theoretical account by using brain imaging and electroencephalogram such as ERPs and SSVEPs (Handy & Mangun, 2000; Parks et al., 2013). When investigating the impact of perceptual load in early visual areas, participants usually performed, at fixation, a main attention-demanding task that varies in perceptual load, while ignoring task-irrelevant information such as high-contrast checkerboards (O'Connor et al., 2002; Schwartz et al., 2005) or motion patterns (Bavelier et al., 2012; Rees et al., 1997) typically presented more peripherally. However, many previous studies focused on either how load affects attentional enhancement or distractor suppression. Here, we investigate how both target enhancement and distractor suppression are concurrently modulated by load by keeping the main task – target and distractors – constant, and rather manipulating attention via cueing and load through the presence or absence of lateral masks.

### Present study

**Fig. 1:**
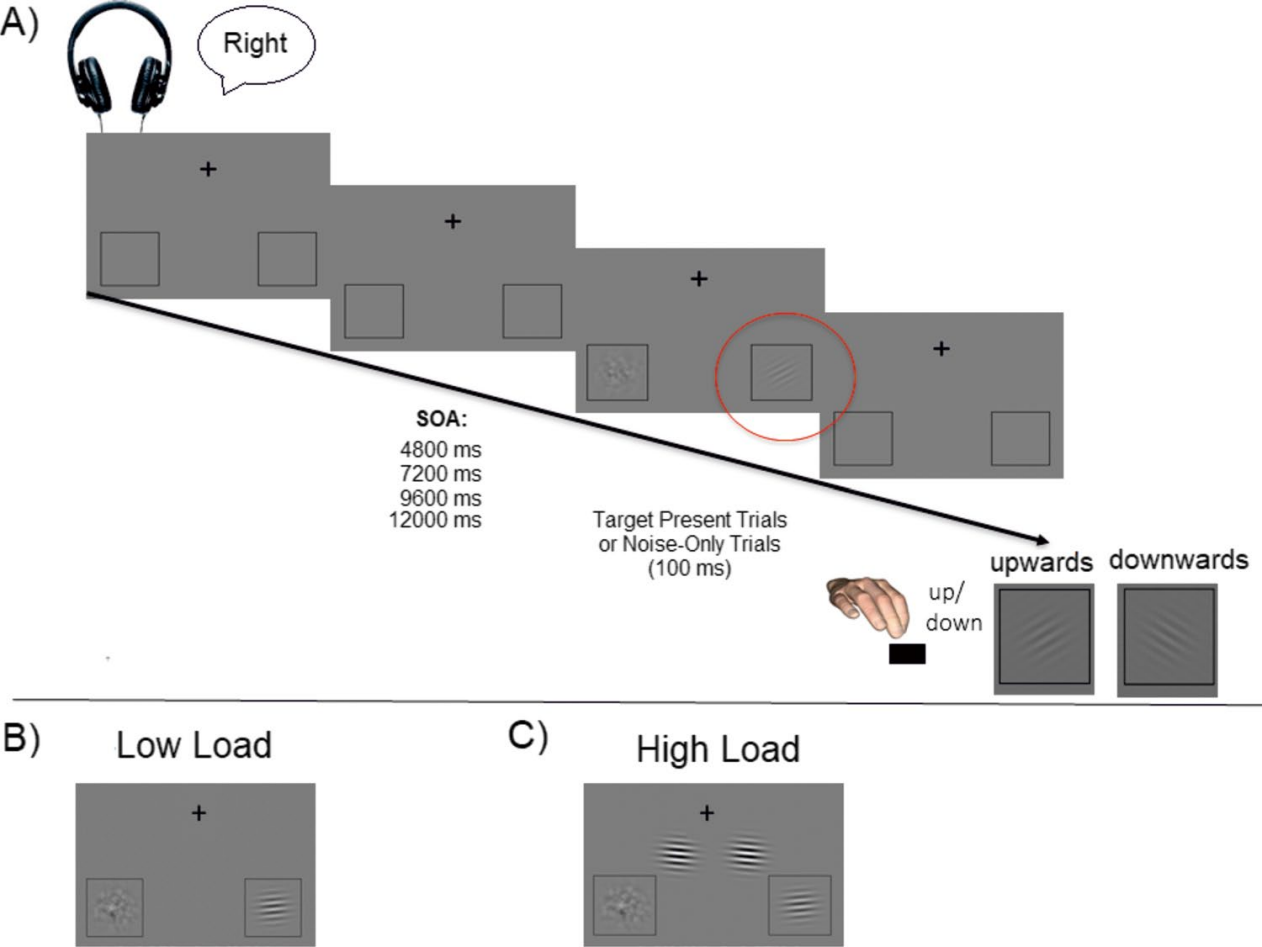
**A** Experimental paradigm. An auditory cue indicated in 75% of the target-present trials the correct (valid) location of the upcoming Gabor patch target. A Gabor patch (target) and a noise patch (distractor) were presented and participants had to respond as fast and as correctly as possible to the orientation of the Gabor patch. **B** Target-present trials kept the main task stimuli constant (a target Gabor patch and a noise patch). **C** In the high-load condition additional high-contrast Gabor patches were presented, acting as lateral masks

In order to further qualify the neural mechanisms of target enhancement and distractor suppression, we presented an adapted Posner cueing paradigm, in which a Gabor patch target was always presented together with a noise patch distractor at two known locations in the left and right visual fields. In each trial, an auditory cue instructed participants to attend to either the left or the right side of the screen (Fig. 1A). The cue was followed by the presentation of the Gabor patch target and the noise patch distractor in 80% of the trials. In these target-present trials, participants were asked to indicate the orientation of the Gabor patch as fast and as correctly as possible and to ignore the noise patch. The auditory cue was predictive (valid) in 60% of the trials and invalid in 20% of the trials. The remaining 20% of the trials consisted of catch trials in which two noise patch distractors were presented and for which participants were asked to withhold their response. This condition will not be discussed further but is described in greater details in Föcker et al. (2018).

Importantly, perceptual load was manipulated through the presentation of two task-irrelevant, high-contrast Gabor patches presented in between the fixation point and the main task stimuli (target Gabor patch and noise patch – see Fig. 1B and C). These high-contrast Gabor patches acted as lateral masks allowing us to increase the perceptual load, while keeping the main task identical. Participants were instructed such lateral masks could be present and to continue reporting the orientation of the target Gabor patch, clearly marked by the locator boxes (Fig. 1C). Such lateral masks were present in 25% of target-present trials.

Thus, this paradigm allows us (1) to investigate neural mechanisms of target enhancement as well as that of distractor suppression, as target and noise patch distractor were either cued or uncued; and (2) to investigate the additional effect of increasing perceptual load on target enhancement and distractor suppression by presenting additional task-irrelevant, salient Gabor patches adjacent to the two possible target areas, acting as lateral masks and thus increasing the main task difficulty (see Fig. 1C).

We expected to find attentional enhancement in early visual areas, that is, a higher activation in early visual areas when the target Gabor patch was cued (valid trials) as compared to uncued (invalid trials). In line with greater target enhancement and distractor suppression under high load, we also expected to see lesser BOLD elicited by the noise patch distractor under high than low load. Of key interest was the impact of attentional status on both target and noise patch distractor-elicited BOLD as a function of load.

## Materials and methods

### Participants

The sample included 32 participants (mean age 21 years; range 18–27 years). Thirty-one participants were right-handed, one participant was left-handed. Participants were recruited at the University of Rochester, New York. This is the identical sample to that reported in Föcker et al. (2018), which consists of both action video game players and non-video game players, and which documented a main effect of perceptual load (distraction) as well a main effect of validity using the behavioural data (inverse efficiency scores as dependent variable), suggesting that the experimental manipulations were successfully applied. For the purpose of this paper, all participants were collated into one group.

All participants had normal or corrected-to-normal visual acuity in both eyes as tested by high-contrast ETDRS format charts with Sloan optotypes (catalog No. 2104; Precision Vision, La Salle, IL, USA). For those participants who needed corrections (four participants), MR-compatible glasses were provided by a trained optometrist. All participants were volunteers who gave written informed consent. The study was approved by the Research Subject Review Board of the University of Rochester.

### Stimuli and task

The stimuli and task have been described in detail in Föcker et al. (2018). In brief, we used an adapted version of the paradigm developed by Sylvester et al. (2007) to measure target enhancement and distractor suppression. As shown in Fig. 1A, each trial started with the presentation of an auditory cue (female voice 500-ms duration saying ‘left’ or ‘right’), which indicated the most likely location of an upcoming Gabor patch target. After a variable stimulus-onset asynchrony (SOA) of 4.8 s, 7.2 s, 9.6 s or 12.0 s, two visual patches (duration of 100 ms) appeared in each of two placeholders. In 80% of the trials, a Gabor patch target was presented on one side and a noise patch on the other side. In 20% of the trials (catch trials) no Gabor appeared (only two noise patches). In 60% of the trials, the cue indicated the correct Gabor location, whereas in another 20% of the trials, participants had to reorient their attention as the Gabor patch was presented at the non-indicated side (the remainder were catch trials). Participants were asked to distinguish between an upwards or a downwards oriented Gabor as fast and as correctly as possible (see Fig. 1A). During catch trials, participants were asked to withhold their response. The next trial started after a variable inter-trial interval (ranging from 2.4 s to 25.2 s) relative to the onset of visual stimuli. Out of the target present trials, 25% were high load (32 out of 128 target present trials in each session), and thus contained two additional irrelevant high-contrast Gabor patches that acted as lateral masks. Overall, 75% validly cued trials were presented (72 out of 96 trials for low load; 24 out of 32 trials for high load in each session) (see Table 1).

**Table 1:** Number of trials in each experimental condition per session and in total

	Low-load valid	Low-load invalid	High-load valid	High-load invalid	Catch trials
Per session	72 trials	24	24	8	32
Total	(2*72) 144	(2*24) 48	(2*24) 48	(2*8)16	(2*32) 64

Throughout the whole experiment, a rotating fixation cross remained on the screen together with two squared boxes in the lower left and right visual fields (4.2° of visual angle from the centre), which served as “landmarks” within which Gabor patch target and/or distractor noise patch distractor always appeared. In order to guarantee fixation, participants were asked to fixate the cross in the centre of the screen and to count the number of missing arms of the fixation cross (65 missing arms were presented across all blocks; block 1: *n* = 7; block 2: *n* = 10; block 3: *n* = 8; block 4: *n* = 9; block 5: *n* = 5; block 6: *n* = 9; block 7: *n* = 9; block 8: *n* = 8). After each block, participants were reported verbally how many missing arms they had counted.

### Experimental design

**Fig. 2:**
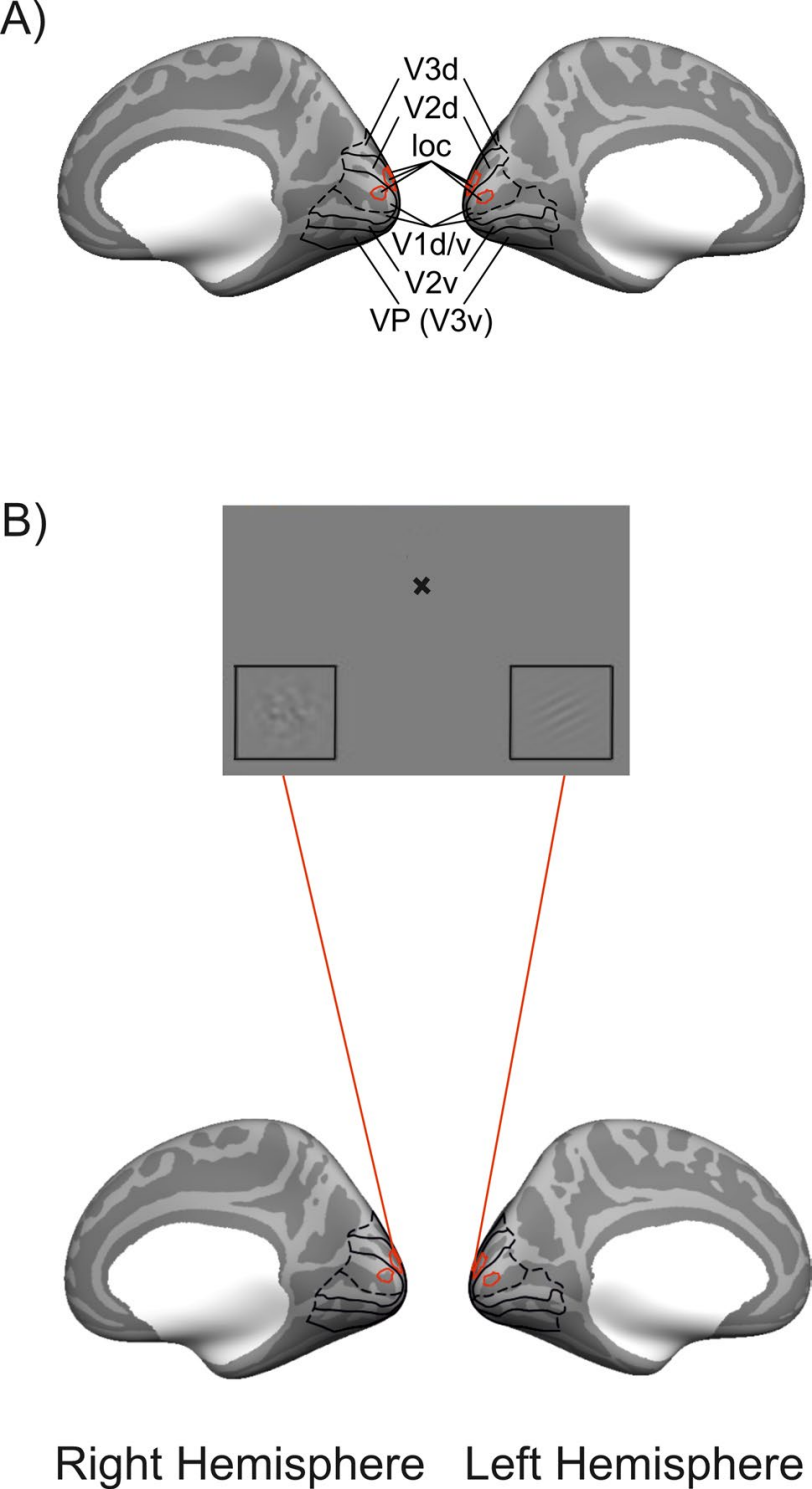
Regions of interest (ROIs) in primary visual areas. **A** Outline of the dorsal (d) and ventral (v) parts of primary (V1), secondary (V2), and tertiary (V3/VP) visual cortex on the (inflated) average brain of Freesurfer. Note that all areas were identified in individual brains. Hence, the outlines indicate those areas that represent the ROIs in the majority of brains. **B** Illustration of one type of task display, here with a target Gabor patch in the right locator box and a noise patch in the left locator box. Blood-oxygenation-level-dependent (BOLD) signal elicited in the corresponding visual area in the hemisphere contra-lateral to the target Gabor patch (respectively the noise patch) was quantified and used as the target Gabor-related (respectively noise patch-related) activation for all analyses

This study consisted of three fMRI sessions – two task-related brain-imaging session as in Fig. 1 (session 1 and session 3) and an intermediate retinotopic mapping and localizer session (session 2, see Fig. 2). Each of those sessions lasted about 1.5 h and were conducted on a separate day.

Prior to the first fMRI session, all participants underwent a training session on our attention task outside the scanner room. The same attention task was repeated during fMRI sessions 1 and 3. However, we adjusted the contrast of the Gabor and noise patches to the 79% threshold of each participant in session 1 and used a fixed contrast (maximum Michelson contrast of .25) in session 3 (see Föcker et al., 2018, for further specifications on the design and stimulus material; in particular the adjustment in contrast aimed to equate for possible differences in contrast sensitivity across action and non-action video game players; see Li, Polat, Scalzo, & Bavelier et al., 2012).

In each fMRI session (1, 3) 160 trials were presented. Among these trials, 96 (60%) were validly cued and 32 trials (20%) were invalidly cued. The remaining 32 trials (20%) consisted of two noise patches and served as catch trials. They were not included in the data analysis. Among the 128 main trials, there were 32 high-load trials, which means that additional high-contrast Gabors were presented next to the target / noise patches. The other 96 trials (without high-contrast Gabors) served as low-load trials (see Table 1).

### MRI acquisition

MRIs were obtained at a Siemens Trio 3T MRI equipped with an eight-channel head coil. The MRIs recorded during the main experiment (see Fig. 1) were acquired as reported in Föcker et al. (2018): Eight fMRI runs (T2*-weighted) were recorded during each session (session 1 and session 3, total 16 runs), with a gradient echo (GE) sequence with echo-planar read-out (EPI) along 36 interleaved axial slices covering the entire brain (TR = 2.4 s, TE = 30 ms, flip angle = 90°, slice thickness = 4 mm, in-plane resolution = 4 × 4 mm^2^, field of view = 256 × 256 mm^2^). A single run consisted of 132–150 volumes (depending on the trial sequence, see above). In order to assure that magnetization reached equilibrium, the trial presentation started after the fifth volume.

During an intermediate session (session 2), five fMRI runs (261 volumes each including five pre-stimulus volumes) were acquired in order to identify early retinotopic visual areas in each individual brain (see below). These were recorded by a GE sequence with EPI read-out along 18 interleaved coronal slices covering the occipital cortex (TR = 1.2 s, TE = 30 ms, flip angle = 90°, slice thickness = 3 mm, in-plane resolution = 3 × 3 mm^2^, field of view = 192 × 192 mm^2^). Additionally, three-dimensional T1-weighted structural images were acquired in each session by a magnetization-prepared, rapid-acquisition gradient-echo (MPRAGE) sequence along 192 sagittal slices (TR = 2530 ms, TE = 3.44 ms, flip angle = 7°, slice thickness = 1 mm, in-plane resolution = 1 × 1 mm^2^, field of view = 256 × 256 mm^2^). Moreover, four fMRI runs (155 volumes each including five pre-stimulus volumes) were recorded along 36 interleaved axial slices covering the entire brain (TR = 2.4 s, TE = 30 ms, flip angle = 90°, slice thickness = 4 mm, in-plane resolution = 4 × 4 mm^2^, field of view = 256 × 256 mm^2^).

### Region of interest (ROI) definition

Regions of interest (ROIs) of relevant visual brain areas (V1, V2, V3) and their visual field representations of the target/noise patch locations (localizer) were identified in individual brains along the cortical surface. Surface-based phase-encoded maps of the polar angle representation of the checkerboards were generated by Freesurfer tools version 4 (Fischl, 2012). For this, the T1-weighted structural image was reconstructed as described previously (Beer et al., 2009). During the reconstruction, a cortical surface at the boundary between white and gray matter was tessellated and automatically registered to a spherical atlas that preserves the individual folding patterns of sulci and gyri (Fischl et al., 1999). Functional images were linearly registered (six degrees of freedom) to the individual T1-weighted image of the reconstruction. Registrations were visually inspected and corrected if necessary. Preprocessing of the functional images included motion correction, intensity normalization, and spatial smoothing (full-width-half-maximum = 5 mm). Polar angle phase maps were calculated by a fast fourier transform (FFT)-type analysis. These maps were projected to the cortical surface of each individual brain and thresholded at a p = 10^-20^. Boundaries between V1, V2, V3/VP and V4 were marked along vertices showing a reversal of the polar phases perpendicular to the calcarine sulcus. Note that the ventral part of V3 (V3v) is sometimes referred to as VP, but our analysis combined both (dorsal and ventral) parts (referred to as V3). The individually defined visual areas were saved as label files and used for subsequent ROI analyses.

Visual field representations of the target/noise patch locations were identified based on the additional visual stimulation runs of session 2. Following preprocessing, which was equivalent to that of the retinotopic runs (see above), the runs were analyzed by a general linear model (GLM). The design matrix included the stimulation protocol (18-s blocks of visual stimulation vs. rest) convolved by a haemodynamic response function (cumulative gamma function). The statistical parametric maps of the contrast Stimulation vs. Rest were then projected to the reconstructed cortical surface of each individual brain and significant regions that overlapped with visual area ROIs were marked. Consequently, ROIs of visual areas (V1, V2, V3) were subdivided in regions representing target/noise patch locations (Localizer) and regions that fall outside this representation (Surround). If the stimuli consisted of a Gabor patch on the right and a noise patch on the left, the target Gabor activity was extracted from the left hemisphere localizer area (respectively its left hemisphere surround for the target surround), and the noise patch activity from the right hemisphere localizer area (respectively its right hemisphere surround for the noise patch surround). Despite remaining controversies about whether ipsilateral fibres may extend to about 1° into the ipsilateral hemifield, the present analyses assume that bottom-up driven visual processing in early visual cortex is essentially limited to stimuli in the contralateral visual field (Zhao et al., 2021). Figure 2 illustrates these ROIs on the group average cortical surface (showing the maximum overlap across subjects).

### ROI analysis

**Fig. 3:**
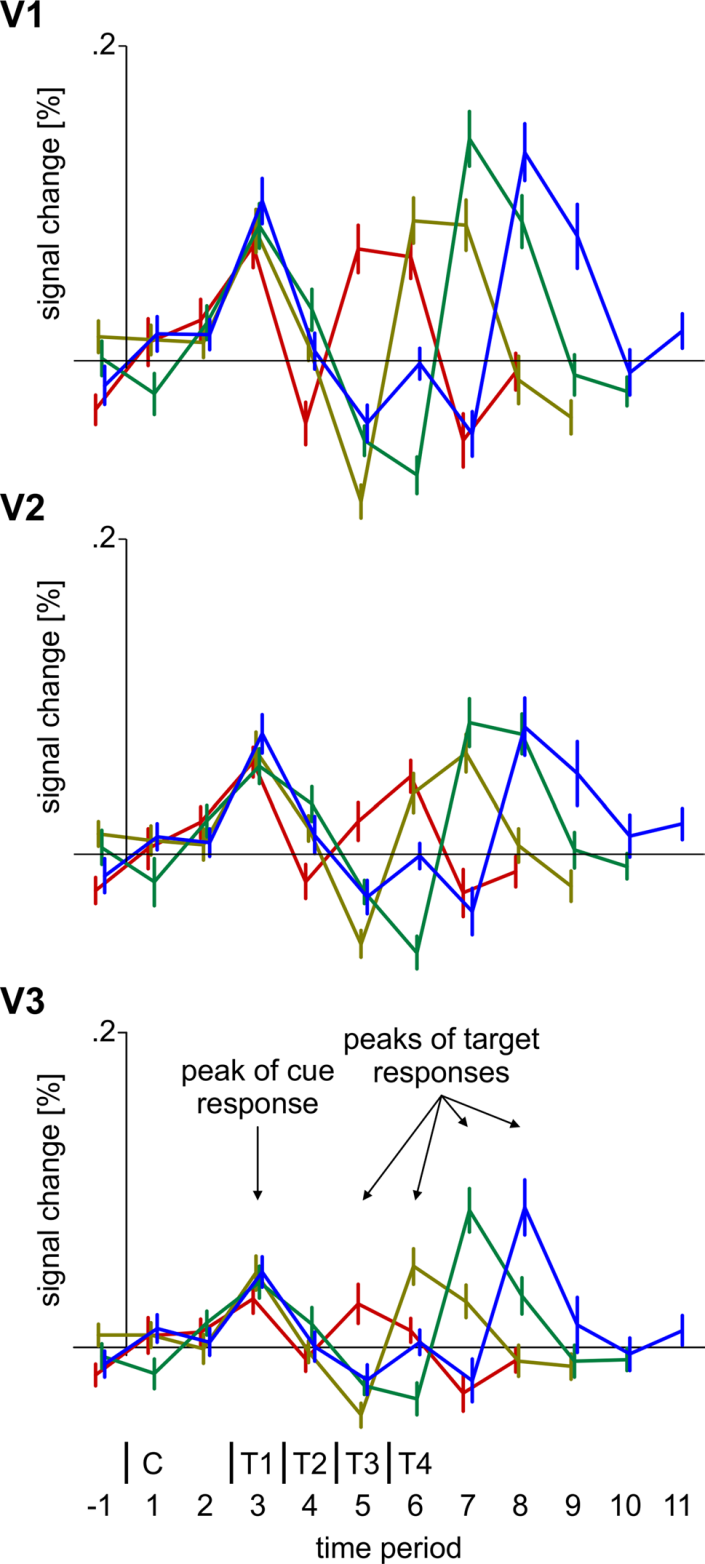
Time course of the blood-oxygenation-level-dependent (BOLD) signal in V1, V2 and V3 shown for each of the four different cue-target periods used (red: T1 = 4.8 s, brown: T2 = 7.2 s, green: T3 = 9.6 s, blue: T4 = 12 s – note that cueing conditions (valid/invalid) are averaged). The time course was extracted by a finite impulse response (FIR) model starting one time period before cue (C) onset. All analyses were performed on the peak of target Gabor patch (respectively noise patch) responses, pooled across the four different cue-target stimulus-onset asynchronies (SOAs)

In order to evaluate target enhancement and distractor suppression in early visual areas, ROI analyses of the BOLD responses to target Gabors and distractor noise patches were conducted. For this, the time course of the BOLD signal was extracted separately for each condition by a GLM analysis from the preprocessed fMRI runs using finite impulse response (FIR) functions as predictors. The FIR design modelled each trial and each time period (measurement) within each trial (overall 12 time periods/TRs including one before trial onset). In addition, estimated head motion parameters and a second-order polynomial per run were added as nuisance regressors in order to model remnant head motion or scanner drift artefacts, respectively. The analysis was performed in native (individual) space. Mean beta-weights were extracted per ROI. Then, beta-weights for each FIR time point were converted into percent signal change (using the pre-trial signal estimate of all conditions as baseline). An illustration of these signal time courses for each of the four different cue-display durations used is provided in Fig. 3 for V1, V2 and V3. Separate time courses were extracted for each condition for each of the six ROIs (V1, V2, V3 by localizer/surround extracted field) and for each hemisphere (contralateral to the target Gabor patch and contralateral to the noise patch). As demonstrated in Fig. 3, the peak response occurred about two time periods after target/noise patch onset and was distinct from the cue-related response. All analyses were performed on the peak response by selecting the BOLD signal two time periods after the target/noise patch onset (see peak of target response in Fig. 3). For the purpose of the present analyses, the BOLD signal was averaged across all four SOA conditions.

### Statistical analysis

#### Behavioural data

A 2 × 2 within-subjects ANOVA was run including the factors *Cue Validity* (valid, invalid) and *Load* (*high versus low*) on reaction times (ms) and accuracy (%). Post hoc t-tests were calculated in order to resolve any interaction effects. Reaction times are reported for correctly identified targets.

#### Brain imaging data

The neural mechanisms of target-enhancement and distractor-suppression were investigated in early visual areas by extracting the BOLD response to the target and to the noise patch from the six ROIs representing the target and noise location (localizer) or the remaining visual hemifield around these locations (surround) separately for V1, V2 and V3. When the target location was cued (valid trials) then the noise patch location was uncued; vice versa, when the target location was uncued (invalid trials) then the noise patch location was cued. The analyses were run for correct trials only. A 2 × 2 × 4 × 3 within-subjects ANOVA including the factors *Cueing* (cued vs. uncued), *Load* (high vs. low load), *Extracted Field* (target localizer and target surround for BOLD elicited on the target side, noise localizer and noise surround for BOLD elicited on the distractor noise patch side) and *Visual Area* (V1, V2, V3) was run on the extracted signal change. Please note that main effects of the factors *Extracted Field* (target localizer and target surround, noise localizer and noise surround) and *Visual Area* (V1, V2, V3) are not easily interpretable given the difference in size of these different ROIs. Results interpretation will therefore focus on the factors *Load* and *Cueing,* as well as possible interactions with *Extracted Field* or *Visual Area*. A Huynh-Feldt correction was applied whenever applicable. Post hoc paired-samples t-tests were calculated in order to resolve any interaction effects.

## Results

### Fixation control task

Overall, participants performed quite well on the central fixation task with an average accuracy of more than 93% (SE = 0.78), indicating that they fixated as instructed most of the time (also reported in Föcker et al., 2018).

### Behavioural results

#### Accuracy

**Fig. 4:**
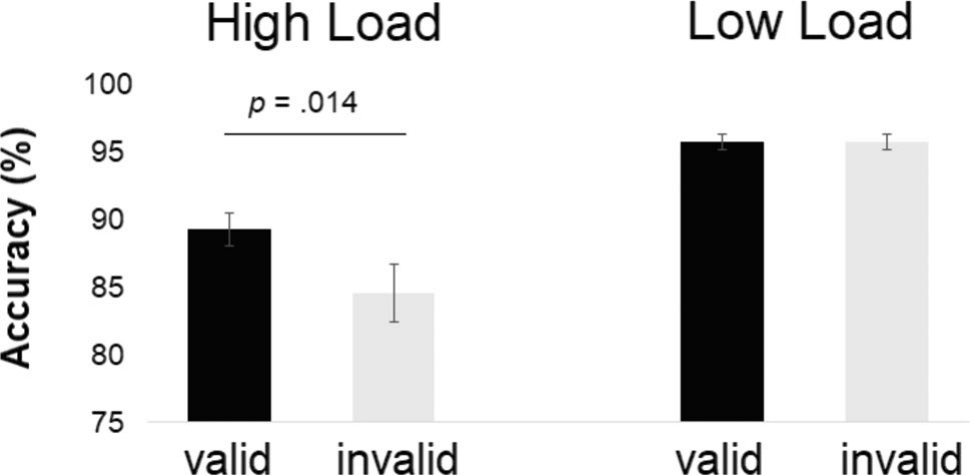
Mean accuracy as a function of cue validity for target-present trials in the high- versus low-load condition

Correct target discrimination (see Fig. 4) was higher in validly cued compared to invalidly cued trials (validly cued *M =* 92%, *SE* = .76; invalidly cued *M =* 89%, *SE* = 1.21; main effect of *Validity*: *F*(1,31) = 6.077, *p* = .019, *ηp*^*2*^ = .164). Furthermore, performance was lower at high load than low load (high *M* = 87%, *SE* = 1.5; low *M* = 96%, *SE* = .47; main effect of *Load*: *F*(1,31) = 44.19, *p* < .001, *ηp*^*2*^ = .588). The interaction between *Validity* and *Load* was also significant showing a higher accuracy for valid than invalid trials at high load, but little validity effects at low load (interaction between *Validity* and *Stimulus Type*: *F*(1,31) = 6.069, *p* = .02, *ηp*^*2*^ = .164; high load valid *M* = 89%; *SE* = 1.24 , invalid *M* = 84%; *SE* = 2.13; *t*(31) = 2.61, *p* = .014, Cohen's d: .461; low load: *p* > .99).

#### Reaction times

**Fig. 5:**
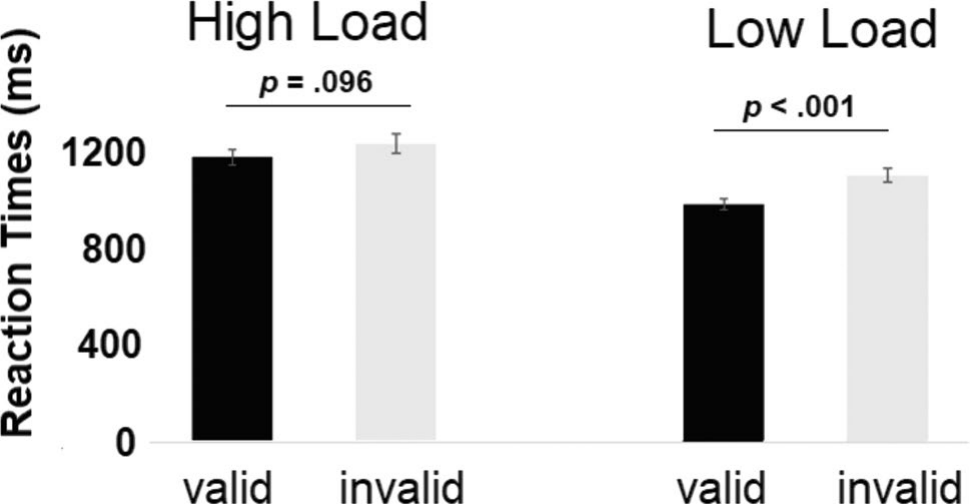
Mean reaction times as a function of cue validity for target-present trials in the high- versus low-load condition

Participants responded faster to validly cued trials (*M* = 1,064 ms; *SE* = 25) compared to invalidly cued trials (*M* = 1,149 ms, *SE* = 30 – main effect of *Validity*: *F*(1,31) = 14.93, *p* = .001, *ηp*^*2*^= .325), and in the low-load (*M* = 1,039 ms, *SE* = 23) as compared to the high-load condition (*M* = 1,174, *SE* = 31; main effect of *Load*: *F*(1,31) = 50.34, *p* < .001, *ηp*^*2*^= .619). The interaction between the factors *Validity* and *Load* was also significant, with faster RTs to valid compared to invalid trials in the low-load condition (valid low load *M* = 982 ms, *SE* = 23; invalid low load *M* = 1,097 ms, *SE* = 27; *t*(31) = -6.89, *p* < .001, Cohen’s d = -1.22), with a less pronounced validity effect in the high-load condition (interaction between *Validity* and *Load*: *F*(1,31) = 5.466, *p* = .02, *ηp*^*2*^ = .150; valid high load *M* = 1,147 ms, *SE* = 32; invalid high load *M* = 1,201 ms, *SE* = 39; *t*(31) = -1.717, *p* = .096, Cohen’s d = -.304). See also Fig. 5.

In all, we note a trade-off between accuracy and RTs as to whether the effect of cueing is most robust on accuracy (high load) or RTs (low load).

#### Inverse efficiency scores

**Fig. 6:**
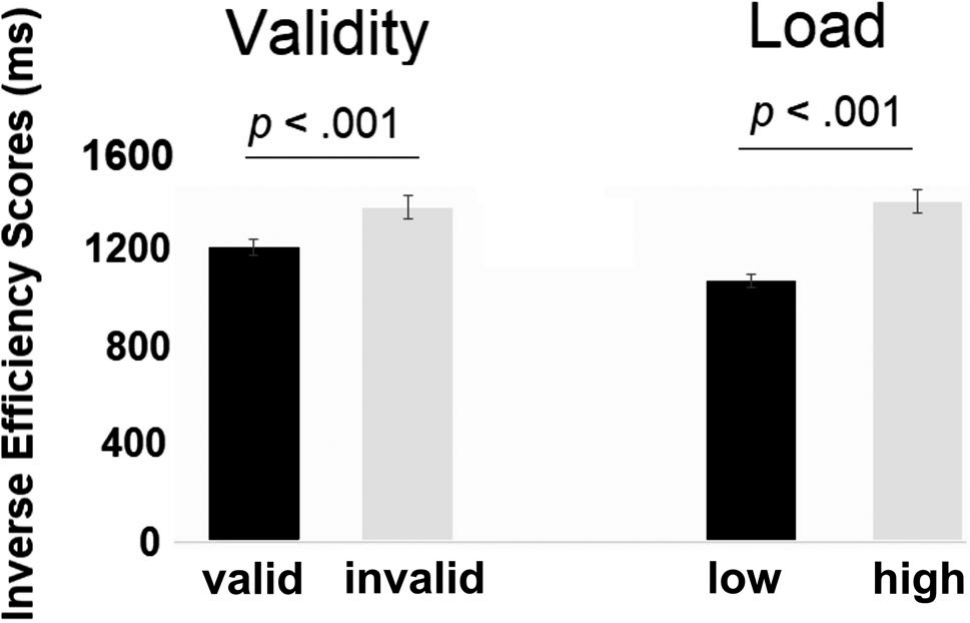
Mean IE scores (ms) as a function of cue validity and load for target-present trials

The analysis of accuracy and RTs suggests a partial trade-off. In order to account for this speed-accuracy trade off, we computed inverse efficiency (IE) scores defined by reaction times divided by (1-error rate) (Bruyer & Brysbaert, 2011; see also Föcker et al., 2018). This IE score was calculated separately for each participant and each condition (low load valid; low load invalid, high load valid, high load invalid). The analysis of the IE scores (see Fig. 6) revealed a significant main effect of *Validity* (*F*(1,31) = 15.104, *p* = .001, *ηp*^*2*^= .328) and a main effect of *Load*: *F*(1,31) = 89.042, *p* < .001, *ηp*^*2*^= .742) suggesting that participants responded more efficiently in validly cued (*M* = 1,170; *SE* = 33) compared to invalidly cued trials (*M* = 1,322; *SE* = 47) and more efficiently in the low-load (*M* = 1,092; *SE* = 25) compared to the high-load condition (*M* = 1,400; *SE* = 48). Importantly, the interaction between *Validity* and *Load* was not significant (*F*(1,31) = 1.328, *p* = .258, *ηp*^*2*^ = .041).

### BOLD response

Bold responses were extracted from three early visual brain areas (V1, V2, V3). All analyses were performed on correct trials only (mean number of correct trials: *M* = 233, *SD* = 7). Each area was subdivided into regions representing the Gabor and noise patch locations (Localizer) and their respective surrounding parts (Surround). First, we examined the BOLD response in ROIs contralateral to the target Gabor patch or in ROIs contralateral to the noise patch as a function of cueing status. We expected to replicate the well-known cueing effect with greater BOLD for cued than uncued targets (*target enhancement*). Of interest was the interaction between cued status and load on target processing. If load modulates target processing, then greater cueing effects from target-related extracted fields were to be expected at high than at low load. With regard to the BOLD responses to the noise patches, we expected reduced responses for cued than uncued patches based on the notion that cued noise patches act as distractors on invalid trials, requiring active re-allocation of attention toward the target and thus disengagement from the wrongly cued distractor noise patch. A Huynh-Feldt correction was applied whenever applicable.

The omnibus 4 × 2 × 2 × 3 ANOVA was run including the factors *Extracted Field* (Target Localizer, Target Surround, Distractor Localizer, Distractor Surround), *Cueing* (cued vs. uncued), *Load* (high vs. low), and *Visual Area* (V1, V2, V3). The main effect of *Cueing* and the main effect of *Load* were not significant (main effect of *Cueing*: *F*(1,31) = .413, *p* = .525, *ηp*^*2*^ *= .013*; main effect of *Load*: *F*(1,31) = .001, *p* = .98, *ηp*^*2*^ *< .001*), nor was the *Load* by *Cueing* interaction (*F*(1,31) = .658, *p* = .424; *ηp*^*2*^ *= .021*). The only significant effect was a *Cueing* by *Extracted Field* interaction (*F*(3,93) = 5.054, *p* = .022, *ηp*^*2*^ *= .14*), which is unpacked below.

#### Cueing effects on target and noise patch processing

**Fig. 7:**
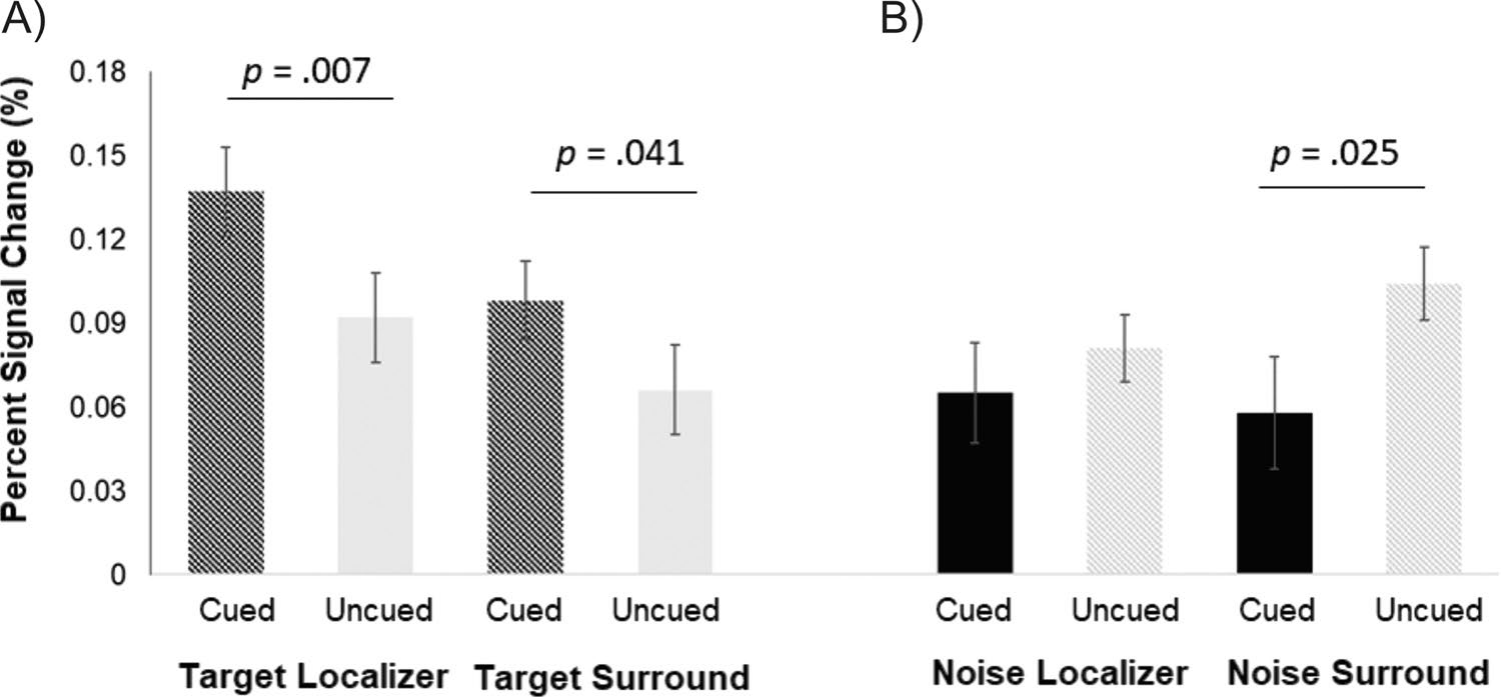
**A** Blood-oxygenation-level-dependent (BOLD) responses elicited by targets extracted either from the patch of cortex contralateral to where the target Gabor patch appeared (Target Localizer) or the surrounding hemifield to this location (Target Surround). **B** BOLD responses elicited by distractor noise patches extracted either from the patch of cortex contralateral to where the distractor noise patch appeared (Noise Localizer) or the surrounding hemifield to this location (Noise Surround). BOLD responses are shown averaged across V1, V2 and V3

The interaction between *Cueing* and *Extracted field* suggests different cueing effects depending on whether the extracted field corresponded to the target or the noise patch distractor. As shown in Fig. 7A, extracted fields contralateral to the Gabor patch target showed a higher BOLD response for cued compared to uncued targets, for both Localizer and Surround extracted fields. This was also confirmed by a subordinate ANOVA, which was run separately for targets, including the factor *Cueing* (cued, uncued) and the factor *Extracted Field* (Localizer, Surround), which indicated a main effect of *Cueing (F*(1,31) = 7.535, *p* = .01, *ηp*^*2*^ *=.*196), due to higher BOLD response for cued (*M* = .117, *SE* = .014) compared to uncued targets (*M* = .079, *SE* = .015).

By contrast, the BOLD response contralateral to the distractor noise patches elicited less BOLD when cued (invalid trials) as compared to uncued (valid trials), with this effect being, if anything, numerically stronger in the surrounding of the distractor noise patch. A subordinate ANOVA that was run separately for distractor noise patches, including the factor *Cueing* (cued, uncued) and the factor *Extracted Field* (Localizer, Surround), indicated that the main effect of *Cueing* was marginally significant (*F*(1,31) = 2.938, *p* = .097, *ηp*^*2*^ *=.*087) with a significant interaction between *Cueing* and *Extracted Field (F*(1,31) = 8.047, *p* = .008, *ηp*^*2*^ =.206*)*, confirming higher cueing effects for the Surround than for the Localizer (cued vs. uncued Noise Localizer: *t*(31) = -.899, *p* = .376, Cohen’s d: -.16; cued vs. uncued Noise Surround: *t*(31) = -2.36, *p* = .025, Cohen’s d: -.42).

#### Load modulation of the cueing effect

**Fig. 8:**
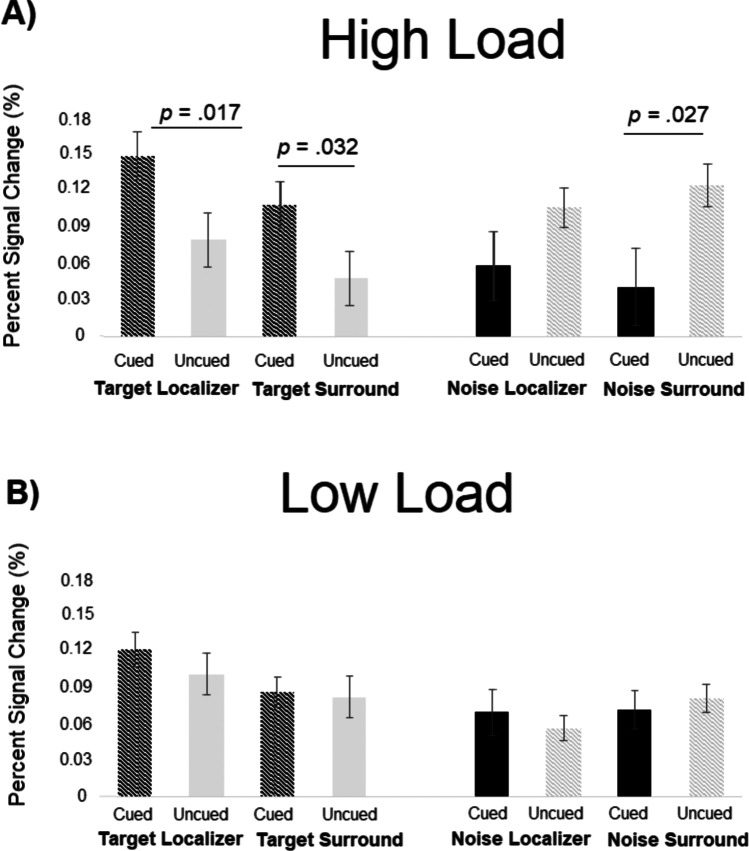
Blood-oxygenation-level-dependent (BOLD) responses extracted from the Target Gabor patch Localizer or its surrounding hemifield and from the distractor Noise patch Localizer or its surrounding hemifield as a function of load: **A** High Load, **B** Low Load

The omnibus ANOVA revealed a marginally significant interaction between the factors *Cueing, Load* and *Extracted Field* (*F*(3,93) = 3.747, *p* = .055; *ηp*^*2*^ = .11), which led us to run separate analyses for high and low load with the factors *Cueing* and *Extracted Field.* The separate analysis for high load revealed a significant interaction between Cueing and Extracted Field (*F*(3,93) = 4.97, *p* = .026, *ηp*^*2*^ = .138). This same interaction was not significant in the low-load condition (*Cueing* by *Extracted Field* interaction: *F*(3,93) = 1.02, *p* = .342, *ηp*^*2*^ = .032). Figure 8A and B illustrate the different impact of being cued or not, which was especially observed at high load, highlighting higher attentional modulation when more selection is required as is the case under high perceptual load.

#### Differences across visual areas

The omnibus ANOVA also indicated several area-specific effects. Main effects of *Visual Area* and of *Extracted Fields*, as well as an interaction between the two, were found (*Visual Area:* V1 *M* = .126, SE = .014 - V2 *M* = .06; SE = .012 - V3 *M* = .076, SE = .015 - *F*(2,62) = 23.12, *p* < .001, *ηp*^*2*^ *= .*427*; Extracted Area*: target localizer region *M =* .114, *SE* = .014; target surround region *M*: .082, *SE* = .013; noise localizer region *M* = .073, *SE* = .012; noise surround area *M=* .091, *SE* = .014 - *F*(3,93) = 10.30, *p* < .001; *ηp*^*2*^ *= *.249 and interaction *F*(6,186) = 3.395, *p* =.038, *ηp*^*2*^ *=* .099). These effects seem to be unrelated to attentional and load modulations and likely reflect variations in the size of the different ROIs.

Of interest, however, are the interactions of *Visual Area* with other factors, especially *Cueing* and *Load*. There was a *Load* by *Visual Area* interaction (*F*(2,62) = 9.502, *p* <.001, *ηp*^*2*^ *=* .235; high load: V1 *M*= .125, SE = .017; V2 *M *= .053, SE = .014; V3 *M* = .084, SE = .016; low load: V1 *M* = .128, SE = .015; V2 *M *= .067, SE = .013; V3 *M *= .068, SE = .017), which appears to reflect greater BOLD at high load compared to low load in V3, whereas V2 and V1 show the opposite pattern or no load effect for both target Gabors and noise patch. However, none of the visual areas showed a significant load effect calling for caution when interpreting this result (V1: *F*(1,31) = .067, *p* = .797, *ηp*^*2*^ *=* .002; V2: *F*(1,31) = 1.038, *p* = .316, *ηp*^*2*^ *=* .032; V3: *F*(1, 31) = 1.345, *p* = .255, *ηp*^*2*^ = .042). There was no interaction between *Visual Area* and *Cueing* (*F*(2,62) = 1.133, *p* =.320, *ηp*^*2*^ *=* .035), nor any triple interactions with Visual Area, Cueing and Load (*F*(2, 62) = .803, *p* = .437, *ηp*^*2*^ = .025).

## Discussion

The main aim of the current experiment was to investigate the neural markers of target enhancement and distractor suppression in early visual areas V1, V2 and V3. Of particular interest were the issues of whether enhancement and suppression effects were increased under the following conditions: (1) when attention was directed either to target or distractor as per cueing, (2) when the task perceptual load was varied.

As expected, participants’ performance was more efficient in validly cued compared to invalidly cued trials suggesting that participants followed the auditory cue instruction and oriented their attention to the side it indicated. Furthermore, and as expected, increased perceptual load impaired task performance: Participants’ response was slower and less accurate at high load than at low load. Although these two factors interacted when analyzing separately reactions times and accuracy, they did not interact when using inverse efficiency scores as a dependent variable. Indeed, while for reaction times cueing effects were only present under low load; for accuracy, cueing effects were only present under high load. This pattern of results suggests different speed-accuracy trade-offs as load is varied, but the overall main effect of cueing remains unchanged across load when considering a more integrated measure of behaviour like the inverse efficiency score.

Our brain imaging results indicated that the BOLD response to the target Gabor patch is enhanced when validly cued as compared to invalidly cued. This was illustrated by greater BOLD contralateral to the Gabor patch location when cued (valid trials) as compared to uncued (invalid trials) across all three visual areas V1, V2 and V3. This is in line with several previous studies documenting target enhancement effects in early visual areas, or in other words a higher BOLD signal, when the stimulus is attended as compared to unattended (Brefczynski & DeYoe, 1999; Corbetta & Shulman, 2002; Halassa & Kastner, 2017; Heinze et al., 1994; Kanwisher & Wojciulik, 2000; Kastner et al.*,* 1998; Kastner & Ungerleider, 2000; O'Connor et al., 2002; Somers et al., 1999; Tootell et al.*,* 1998).

Importantly, by varying perceptual load, we could establish that the target enhancement effect or the difference in BOLD signal contralateral to the target Gabor when cued versus uncued is most robust under high perceptual load. This effect is in line with previous electrophysiological studies demonstrating a higher N1 in high-load compared to low-load conditions as well as higher attentional effects under high- versus low-load conditions (Awh & Pashler, 2000; Handy & Mangun, 2000).

Thanks to the present design, it was also possible to monitor the fate of the distractor noise patch always presented along the target Gabor, by looking at the BOLD signal elicited in the hemisphere contralateral to that noise patch. The BOLD signal elicited by cued noise patches (invalid trials) was lower than that elicited by uncued noise patches (valid trials). As with targets, this effect was especially marked in the high-load condition. Note that in the present design, the main task stimuli – target Gabor patch and noise patch – remain identical as perceptual load is varied through the presence/absence of lateral masks. This design thus allows to monitor the distribution of attention to main task stimuli as a function of both their task relevance and load.

**Fig. 9:**
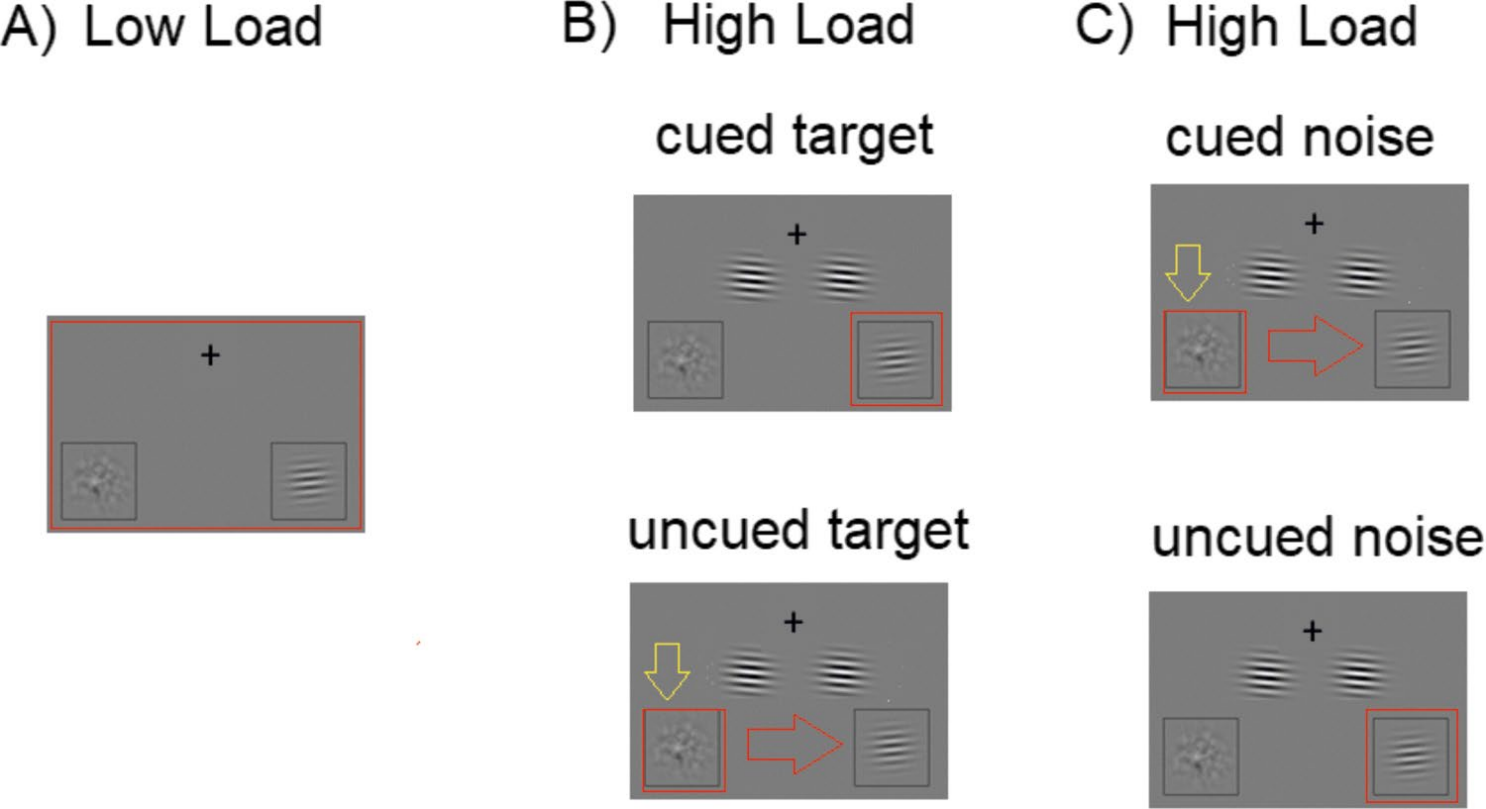
**A** The attentional window, schematized by the red box, is broad under low load. **B, C** Under high load (lateral masks present), the attentional focus narrows down to the location announced by the auditory cue. If the target happens to be presented at that cued location, no movement of attention is needed (see ‘cued target’ and ‘uncued noise’, which captures the fate of target and distractor noise patches in validly cued trials). If the noise patch happens to be presented at that cued location, then a movement of attention is needed (see ‘uncued target’ and ‘cued noise’, which captures the fate of target and distractor noise patches in invalidly cued trials). In this latter case, the yellow arrow demonstrates the required suppression of the distractor noise patch during the reorientation process

A possible interpretation, in line with the load theory of Lavie (1995), is that, upon presentation of a low-load display, the attentional window tends to widen encompassing not only target but also some of the noise patch. This large attentional window means target and surround locations are processed and, consequently, a reduced modulation of the BOLD response is observed between cued and uncued targets as well as cued and uncued distractor noise patch (see Fig. 8B). In contrast, upon presentation of a high-load display that contains salient stimuli in the surround region, the attentional window tends to narrow onto the localizer position that has been referred to by the auditory cue. If the target is present at this position, it will benefit from this attentional focus; however, if the distractor noise patch is present, suppression will be needed to allow for a move of attention away from this position and toward the target location to perform the task correctly. Taken together, this may account for stronger modulations of the BOLD response between cued (valid) and uncued (invalid) trials under high load as compared to low load. See also Fig. 9 for a schematic illustration.

Additionally, the interaction between *Cueing* and *Extracted Field* seen at high load reveals that the impact of cued status on the target-elicited BOLD tends to be greater in the Localizer than in the Surround area, whereas the reverse tends to be observed with noise patch-elicited BOLD. Indeed, at high load, the impact of cued status on the noise patch-elicited BOLD tends to be, if anything, numerically greater in the Surround than in the Localizer extracted field. While the presentation of highly perceptually salient stimuli in the Surround could explain the latter effect, it remains that the Localizer region is the one to show the greatest BOLD modulation with cued status when considering target-elicited BOLD. A major distinction is that when the target is validly cued, the initial allocation of attention as a result of the cueing can remain in place. In contrast, when the noise patch location is cued, albeit invalidly, the noise patch side needs to be suppressed as attention disengages to move to the target side. The present pattern of results suggests this suppression mechanism is quite broad. Under high load, it seems to include the Surround region, likely owing to the presence of salient stimuli in the Surround in that condition. Moreover, the overall greater BOLD seen in valid trials as compared to invalid trials points again toward a more global suppression in these early visual areas when attention has to be re-allocated after having been wrongly cued, with this rather generalized suppression effect being again stronger at high load than low load.

## Methodological considerations

The current experimental design does not follow strictly the experimental design, as initially used by Lavie and collaborators, to investigate the distribution of attention as perceptual load varies (Lavie, 1995). In the standard perceptual load design, the main task difficulty is manipulated by complexifying the stimuli that are part of the main task (e.g., contrasting a visual search among homogenous versus heterogenous search items), while monitoring the fate of distractors. The distractors are thus kept constant, and the stimuli that are part of the main task manipulated. In this fashion, one can show that distractors are easier to ignore under the high perceptual load condition compared to the low perceptual load condition. In our current experimental design, the main task stimuli remain identical as perceptual load is manipulated by the presence/absence of lateral masks. In this way, we can track the processing of the stimuli that form the main task as a function of attention allocation, which was varied by a load manipulation as well as a cueing manipulation in the present study.

## Conclusion

To conclude, our findings suggest that enhancement of task-relevant information but also the suppression of irrelevant information in early visual areas is increased under high perceptual load. As previously reported, cueing to a specific location in space guarantees that higher attentional resources are available to increase processing at this location if it contains task relevant information. On the other hand, if such cueing is invalid, the present work documents a rather generalized suppression of not just the cued location but also the cortex in the surrounding hemifield. In accordance with Lavie’s theory of visual attention, these effects were most pronounced at high perceptual load. Future studies might aim to look into the interaction between the frontoparietal network, and in particular the frontal eye field given its major role in attention allocation, and such rather broad distractor suppression as attention becomes properly reallocated in order to further characterize the source of such suppression effect.

## Data Availability

The datasets generated during and/or analyzed during the current study are available on reasonable request.
